# Comparative speed of kill of sarolaner (Simparica^®^) and afoxolaner (NexGard^®^) against induced infestations of *Ixodes holocyclus* on dogs

**DOI:** 10.1186/s13071-017-2024-9

**Published:** 2017-02-21

**Authors:** Raj Packianathan, Andrew Hodge, Natalie Bruellke, Kylie Davis, Steven Maeder

**Affiliations:** 1 Zoetis Australia Research and Manufacturing Pty Ltd, Veterinary Medicine Research and Development, Level 6, 5 Rider Boulevard, Rhodes, NSW 2138 Australia; 2 Zoetis, Veterinary Medicine Research and Development, 333 Portage St, Kalamazoo, MI 49007 USA

**Keywords:** *Ixodes holocyclus*, Tick, Dog, Sarolaner, Simparica^®^, Afoxolaner, NexGard^®^, Isoxazoline, Oral, Speed of kill

## Abstract

**Background:**

The Australian paralysis tick, *Ixodes holocyclus,* causes paralysis predominantly in dogs and cats in the Eastern coastal regions of Australia. Rapid onset of effect of a parasiticide is critical to minimize the deleterious effects of these tick infestations, especially tick paralysis caused by the salivary neurotoxin. The speed of kill of a novel orally administered isoxazoline parasiticide, sarolaner chewable tablets (Simparica^®^), against *I. holocyclus* on dogs was evaluated and compared with afoxolaner (NexGard^®^) for 5 weeks after a single oral dose.

**Methods:**

Twenty-four (24) dogs were randomly allocated to treatment with either placebo, sarolaner (label dose of 2 to 4 mg/kg as per dosing table), or afoxolaner (label dose of 2.7 to 6.9 mg/kg) based on pre-treatment body weights. Following artificial infestation on Day -1, dogs were examined and live ticks counted at 8, 12, 24 and 48 h after treatment on Day 0, and at 12, 24 and 48 h after subsequent re-infestations on Days 7, 14, 21, 28 and 35. Efficacy was determined at each time point relative to counts for placebo dogs based on geometric means.

**Results:**

At 8 and 12 h time points on Day 0, sarolaner-treated dogs had significantly lower geometric mean tick counts compared to the dogs treated with afoxolaner (*P* ≤ 0.0303). Efficacy of sarolaner against an existing infestation was 86.2 and 96.9% compared with that of afoxolaner which had efficacy of 21.3 and 85.0% at 8 and 12 h time points, respectively. Against subsequent weekly re-infestations at 12 h time points, treatment with sarolaner resulted in significantly lower geometric mean tick counts than afoxolaner-treated dogs on all days (*P* ≤ 0.0077) with the efficacy ranging from 60.2 to 92.2%, compared to 5.8 to 61.0% in the afoxolaner-treated dogs. Against subsequent weekly re-infestations at the 24 h time points on Days 22 and 36, efficacy of sarolaner was significantly higher at 99.2 and 97.9%, respectively, compared with afoxolaner which had efficacy of 92.4 and 91.9% (*P* ≤ 0.0356*)*. At the 48 h time points following each of the five weekly re-infestations, the mean efficacy results of sarolaner and afoxolaner treated dogs were similar on most occasions. There were no adverse reactions to treatments.

**Conclusions:**

In this controlled laboratory evaluation, a single dose of sarolaner had a significantly faster speed of kill against an existing infestation of *I. holocyclus*, than afoxolaner at 8 and 12 h post-treatment. The rapid and consistent kill of ticks provided by sarolaner within 24 h after a single oral dose and following weekly re-infestations over 35 days suggests this treatment will provide highly effective, rapid and reliable control of ticks over the entire treatment interval, thereby minimizing the risk of tick paralysis in dogs.

**Electronic supplementary material:**

The online version of this article (doi:10.1186/s13071-017-2024-9) contains supplementary material, which is available to authorized users.

## Background

*Ixodes holocyclus*, also known as the paralysis tick in Australia, causes tick paralysis in domestic animals, a potentially-lethal disease seen predominantly in dogs and cats [1–3], horses [4–6] and humans [7–9]. *Ixodes holocyclus* is widely distributed along the eastern coastal regions of Australia from North Queensland to Lakes Entrance of Victoria [1, 10–12]. The majority of cases of tick paralysis in dogs are reported during spring to early summer due to favorable climate [1, 11]. *Ixodes holocyclus* is suspected as a vector for transmission of *Borrelia burgdorferi* (*sensu stricto*) [13] and *Rickettsia australis* [14, 15] in humans, but it is rarely reported as a vector for transmission of other pathogens in domestic animals.

Tick paralysis is characterised by progressive ascending lower motor neuron (LMN) flaccid paralysis caused by the neurotoxin produced in the salivary glands of the female *I. holocyclus* [3, 16]. Clinical manifestations of tick paralysis have been previously reviewed and well documented and include flaccid paralysis, cardio-pulmonary complications and sometimes death [3, 17, 18]. Typically, the onset of clinical signs usually does not occur until 4 or 5 days after tick attachment [3, 17, 19]. This coincides with a sudden increase in the salivary toxin secretions which occur during the later stages of tick infestations [3, 16]. The duration of feeding, rate of growth and release of toxins however can vary depending on the age of the ticks and favorable ambient temperature and humidity [20]. The longer the duration of attachment, the greater the potential risk of paralysis, thus the sooner an attached tick can be removed, the lower the risk of paralysis [17, 19]. Therefore, an acaricide with a fast speed of kill is critical in removing any pre-existing ticks and minimising the risk of morbidity and mortality due to tick paralysis [21].

Treatment of tick paralysis typically centers around removal of attached ticks, the neutralization of toxins, the control of clinical manifestations of tick paralysis and the treatment of any anaphylactic reactions due to the administration of tick anti-sera [22]. Successful treatment outcomes also depend on locating and removing all the attached ticks on the dogs followed by the administration of an acaricidal agent [23]. A routine tick search, even by an experienced person, may be unsuccessful in locating all ticks, even in the common predilection sites [20], since ticks can be hidden in areas such as the nostril, inside the anus and ear canals, hence an acaricide with a wider systemic distribution should be the preferred choice of treatment. Systemic formulations like the new class of isoxazolines offer wider distribution to all anatomical sites in the body [21] and are therefore able to provide more reliable efficacy, unlike the topical counterparts [23] which may not reach all the anatomical sites.

The introduction of isoxazolines such as sarolaner, afoxolaner and fluralaner has resulted in an improvement in tick control in dogs in recent times. Sarolaner, a pure form of S-entamioner, is a potent ectoparaciticide [24] with a broad spectrum of activity against different tick species [25, 26], including the Australian paralysis tick, *I. holocyclus* (Zoetis, unpublished data).

In this laboratory study, the speed of kill of sarolaner (Simparica^®^) and afoxolaner (Nexgard^®^) was evaluated against existing *I. holocyclus* infestations and weekly re-infestations for a period of 5 weeks after treatment with a single dose.

## Methods

The study was a blinded, negative-controlled, randomized laboratory efficacy design conducted in New South Wales, Australia. Study procedures were in accordance with the World Association for the Advancement of Veterinary Parasitology (WAAVP) guidelines for evaluating the efficacy of parasiticides for the treatment, prevention and control of flea and tick infestation on dogs and cats [27] and complied with the principles of Good Clinical Practice [28]. The protocol was reviewed and approved by the Vet^x^ Research Animal Ethics Committee, NSW. Blinding of the study was assured through the separation of functions. All personnel conducting observations, or performing infestations and counts were blinded to treatment allocation.

### Animals

Twenty-four (24) male and female, Foxhound dogs from 1 to 9 years of age and weighing from 30.1 to 46.9 kg were used in the study. Each dog was individually identified by a unique electronic transponder and had undergone an adequate wash-out period to ensure that no residual ectoparasiticide efficacy remained from any previously administered treatments. This was confirmed by the tick carrying capacity test results that showed all enrolled dogs had ≥ 21 ticks. Dogs were individually housed in indoor runs such that no physical contact was possible between them and they were acclimatized to these conditions for at least 7 days prior to treatment. Dogs were fed an appropriate maintenance ration of a commercial dry canine feed for the duration of the study. Water was available *ad libitum*. All dogs were given a physical examination to ensure that they were in good health at enrollment and suitable for inclusion in the study. General health observations were performed twice daily throughout the study.

### Design

The study followed a randomized complete block design, with pairs of dogs as the experimental unit. Dogs enrolled in the study were immunised to the tick toxin- holocylotoxin as described previously [29] and selected based on acceptable results (tick counts of  ≥ 21) to the tick carrying capacity test performed on Day -7. Prior to treatment, dogs were ranked according to pre-treatment body weights into four blocks of six (three pairs of dogs). Within each block, one pair of dogs was randomly allocated to treatment with placebo, sarolaner, or afoxolaner. There were eight dogs per treatment group.

### Treatment

Body weights collected on Day -5 were used to determine the appropriate dose to be administered. On Day 0, dogs received either a placebo tablet, Simparica^®^ (sarolaner) at the recommended dose of 2 mg/kg (range: 2 to 4 mg/kg), or NexGard^®^ (afoxolaner) as per label directions (2.7 to 6.9 mg/kg). All doses were administered by hand pilling to ensure accurate and complete dosing. Each dog was observed for at least 2 min after treatment to ensure the dose was swallowed.

### Tick infestation and assessment

The unfed adult female *I. holocyclus* ticks were collected from the Northern Rivers region of New South Wales, Australia approximately 2 months prior to the start of the study. The ticks were stored in dark conditions at around 12 °C and high humidity [29]. Tick infestations were performed on Days -7 (host suitability), -1, 7, 14, 21, 28 and 35. Prior to each infestation, dogs were examined to ensure they were free of ticks. Each dog was infested with 30 viable unfed female ticks at pre-defined locations (head, shoulders, dorsal midline of the body) on the dogs as described previously [29]. For Day -1 infestation, tick counts were performed at 8, 12, 24 and 48 h after treatment on Day 0 and all other tick counts were performed at 12, 24 and 48 h after each weekly infestation. Tick assessments at 8 (only on Day 0), 12 and 24 h time points were performed without removing the ticks from the dogs. After counting at the 48 h time point, all ticks were removed. Ticks were characterised as described previously [29] except moribund ticks were recorded in a separate category and included in the live counts in this study for the efficacy calculations.

### Statistical analysis

The primary outcome measure was live tick counts. Data for post-treatment live (free plus attached) tick counts were summarized with arithmetic (AM) and geometric (GM) means by treatment group and time point. Tick counts were transformed by the log_e_(count + 1) transformation prior to analysis in order to stabilize the variance and normalize the data. Using the PROC MIXED procedure (SAS 9.3, SAS Institute Inc., Cary, NC, USA), transformed counts were analyzed using a mixed linear model for repeated measures for the 12, 24 and 48 h time points separately. The fixed effects were treatment, time point and the interaction between time point and treatment. The random effects included block, pair, animal, block by treatment by time point interaction, and error. The data for the 8 h time point (Day 0 only) were analysed with terms including the fixed effect of treatment group and the random effects of block, pair and error. Testing was two-sided at the significance level α = 0.05, with tests based on contrasts between treatment least squares means from the fitted models.

The assessment of efficacy for live ticks was based on the percent reduction in the AM and GM live tick counts for the treated groups relative to placebo, as suggested by the most recent guidelines of the WAAVP for systemic acaricides [27], and was calculated using Abbott’s formula:

$$ \%\ \mathrm{reduction}=100 \times \frac{\mathrm{mean}\ \mathrm{count}\ \left(\mathrm{placebo}\right)-\mathrm{mean}\ \mathrm{count}\ \left(\mathrm{treated}\right)}{\mathrm{mean}\ \mathrm{count}\ \left(\mathrm{placebo}\right)} $$

As the distribution of parasite counts within each group was likely be skewed, comparison between groups was primarily based on GM live tick counts [27].

## Results

There were no treatment-related adverse events during the study. Placebo-treated dogs maintained good tick infestations throughout the study with individual tick counts ranging from 15 to 30 (Tables 1, 2, 3 and 4).

**Table 1:** Mean live *Ixodes holocyclus* counts and efficacy relative to placebo at 8, 12, 24 and 48 h after treatment for dogs treated with a single oral dose of sarolaner or afoxolaner on Day 0

Treatment		Tick counting time points post-treatment			
		8 h	12 h	24 h	48 h
Placebo	Range	21–28	20–28	19–28	19–30
	Arithmetic mean (AM)	24.13	24.25	23.63	23.75
	Geometric mean GM)^a^	24.00	24.13	23.47	23.53
Sarolaner	Range	0–13	0–8	0–0	0–0
	Arithmetic mean (AM)	5.13	1.63	0	0
	AM Efficacy (%)	78.87	93.30	100	100
	Geometric mean (GM)^a^	3.32	0.76	0	0
	GM Efficacy (%)	86.17	96.87	100	100
	Test statistic *vs* placebo	*t*_(9)_ = 5.02	*t*_(8)_ = 8.65	*t*_(34)_ = 17.31	*t*_(17)_ = 58.45
	*P*-value *vs* placebo	0.0007	< 0.0001	< 0.0001	< 0.0001
Afoxolaner	Range	10–28	1–13	0–2	0–0
	Arithmetic mean (AM)	20.13	5.00	0.38	0
	AM Efficacy (%)	17.01	79.38	98.41	100
	Geometric mean (GM)^a^	18.89	3.63	0.25	0
	GM Efficacy (%)	21.28	84.95	98.93	100
	Test statistic *vs* placebo	*t*_(9)_ = 0.65	*t*_(8)_ = 6.24	*t*_(46)_ = 11.78	*t*_(17)_ = 58.45
	*P*-value *vs* placebo	0.5302	0.0003	< 0.0001	< 0.0001
	Test statistic *vs* sarolaner	*t*_(9)_ = 4.36	*t*_(15)_ = 2.40	*t*_(70)_ = 0.74	*t*_(17)_ = 0.00
	*P*-value *vs* sarolaner	0.0018	0.0303	0.4622	1.0000

^a^ *P*-values are based on comparison of geometric means

**Table 2:** Mean live *Ixodes holocyclus* counts and efficacy relative to placebo at 12 h after weekly re-infestations for dogs treated with a single oral dose of sarolaner or afoxolaner on Day 0

Treatment		Day of study				
		7	14	21	28	35
Placebo	Range	16–28	22–28	17–26	19–27	23–28
	Arithmetic Mean (AM)	21.75	25.25	23.75	23.13	25.13
	Geometric Mean (GM)^a^	21.44	25.16	23.56	22.99	25.09
Sarolaner	Range	0–12	0–10	0–18	2–21	2–16
	Arithmetic mean (AM)	3.00	3.00	4.88	10.75	8.75
	AM Efficacy (%)	86.21	88.12	79.47	53.51	65.17
	Geometric mean (GM)^a^	1.67	2.26	3.11	9.15	7.50
	GM Efficacy (%)	92.20	91.03	86.78	60.21	70.11
	Test statistic *vs* placebo	*t*_(8)_ = 6.26	*t*_(8)_ = 8.50	*t*_(8)_ = 5.41	*t*_(9)_ = 3.77	*t*_(8)_ = 4.97
	*P*-value *vs* placebo	0.0002	< 0.0001	0.0007	0.0048	0.0012
Afoxolaner	Range	2–16	7–20	10–22	15–28	16–27
	Arithmetic mean (AM)	9.63	12.13	17.63	22.00	21.00
	AM Efficacy (%)	55.75	51.98	25.79	4.86	16.42
	Geometric mean (GM)^a^	8.36	11.49	17.03	21.66	20.78
	GM Efficacy (%)	61.01	54.31	27.69	5.78	17.17
	Test statistic *vs* placebo	*t*_(9)_ = 3.85	*t*_(10)_ = 6.26	*t*_(15)_ = 2.65	*t*_(17)_ = 0.67	*t*_(15)_ = 2.30
	*P*-value *vs* placebo	0.0042	< 0.0001	0.0182	0.5138	0.0366
	Test statistic *vs* sarolaner	*t*_(13)_ = 3.16	*t*_(10)_ = 5.07	*t*_(9)_ = 4.37	*t*_(9)_ = 3.44	*t*_(8)_ = 4.10
	*P*-value *vs* sarolaner	0.0077	0.0004	0.0021	0.0070	0.0033

^a^ *P*-values are based on comparison of geometric means

**Table 3:** Mean live *Ixodes holocyclus* counts and efficacy relative to placebo at 24 h after weekly re-infestations for dogs treated with a single oral dose of sarolaner or afoxolaner on Day 0

Treatment		Day of study				
		8	15	22	29	36
Placebo	Range	17–27	20–28	15–26	19–26	19–26
	Arithmetic Mean (AM)	21.00	24.13	23.75	22.25	22.88
	Geometric Mean (GM)^a^	20.70	23.95	23.44	22.13	22.78
Sarolaner	Range	0–6	0–1	0–3	0–3	0–3
	Arithmetic mean (AM)	1.75	0.25	0.38	1.25	0.75
	AM Efficacy (%)	91.67	98.96	98.42	94.38	96.72
	Geometric mean (GM)^a^	1.26	0.19	0.19	0.96	0.49
	GM Efficacy (%)	93.93	99.21	99.19	95.67	97.86
	Test statistic *vs* placebo	*t*_(34)_ = 12.26	*t*_(34)_ = 16.48	*t*_(34)_ = 16.37	*t*_(34)_ = 13.37	*t*_(34)_ = 15.01
	*P*-value *vs* placebo	< 0.0001	< 0.0001	< 0.0001	< 0.0001	< 0.0001
Afoxolaner	Range	0–5	0–1	0–6	0–5	0–8
	Arithmetic mean (AM)	2.00	0.25	2.75	1.75	2.75
	AM Efficacy (%)	90.48	98.96	88.42	92.13	87.98
	Geometric mean (GM)^a^	1.41	0.19	1.78	1.14	1.85
	GM Efficacy (%)	93.17	99.21	92.42	94.87	91.89
	Test statistic *vs* placebo	*t*_(46)_ = 8.70	*t*_(46)_ = 12.06	*t*_(46)_ = 8.62	*t*_(46)_ = 9.44	*t*_(46)_ = 8.41
	*P*-value *vs* placebo	< 0.0001	< 0.0001	< 0.0001	< 0.0001	< 0.0001
	Test statistic *vs* sarolaner	*t*_(70)_ = 0.22	*t*_(70)_ = 0.00	*t*_(70)_ = 2.80	*t*_(70)_ = 0.29	*t*_(70)_ = 2.14
	*P*-value *vs* sarolaner	0.8247	1.0000	0.0066	0.7757	0.0356

^a^ *P*-values are based on comparison of geometric means

**Table 4:** Mean live *Ixodes holocyclus* counts and efficacy relative to placebo at 48 h after weekly re-infestations for dogs treated with a single oral dose of sarolaner or afoxolaner on Day 0

Treatment		Day of study				
		9	16	23	30	37
Placebo	Range	17–26	20–28	15–26	21–27	19–26
	Arithmetic mean (AM)	21.00	24.13	22.00	22.88	22.25
	Geometric mean (GM)^a^	20.79	23.98	21.69	22.80	22.15
Sarolaner	Range	0–0	0–2	0–2	0–3	0–1
	Arithmetic mean (AM)	0	0.38	0.50	1.25	0.25
	AM Efficacy (%)	100	98.45	97.73	94.54	98.88
	G. mean (GM)^a^	0	0.25	0.32	1.03	0.19
	GM Efficacy (%)	100	98.95	98.54	95.48	99.15
	Test statistic *vs* placebo	*t*_(19)_ = 54.50	*t*_(25)_ = 20.30	*t*_(19)_ = 13.13	*t*_(23)_ = 12.78	*t*_(26)_ = 21.67
	*P*-value *vs* placebo	< 0.0001	< 0.0001	< 0.0001	< 0.0001	< 0.0001
Afoxolaner	Range	0–0	0–1	0–4	0–2	0–1
	Arithmetic mean (AM)	0	0.13	1.50	0.38	0.25
	AM Efficacy (%)	100	99.48	93.18	98.36	98.88
	G. mean (GM)^a^	0	0.09	1.28	0.25	0.19
	GM Efficacy (%)	100	99.62	94.12	98.90	99.15
	Test statistic *vs* placebo	*t*_(19)_ = 54.50	*t*_(25)_ = 21.23	*t*_(19)_ = 10.60	*t*_(23)_ = 15.29	*t*_(26)_ = 21.67
	*P*-value *vs* placebo	< 0.0001	< 0.0001	< 0.0001	< 0.0001	< 0.0001
	Test statistic *vs* sarolaner	*t*_(19)_ = 0.00	*t*_(25)_ = 0.93	*t*_(19)_ = 2.53	*t*_(23)_ = 2.51	*t*_(26)_ = 0.00
	*P*-value *vs* sarolaner	1.0000	0.3606	0.0204	0.0195	1.0000

^a^ *P*-values are based on comparison of geometric means

**Fig. 1:**
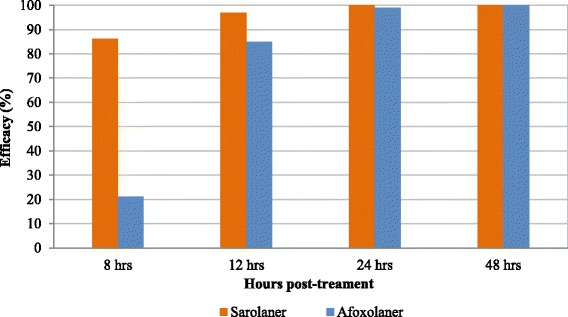
Percent efficacy based on geometric mean counts relative to placebo at 8, 12, 24 and 48 h after treatment for dogs treated with a single oral dose of sarolaner or afoxolaner on Day 0

Against an existing infestation, at the 8 and 12 h time points, treatment with sarolaner resulted in significantly lower GM tick counts compared to both placebo (*P* ≤ 0.0007) and afoxolaner treated dogs (*P* ≤ 0.0303). There was no significant difference between the GM live tick counts at 8 h for the afoxolaner and placebo-treated dogs (*P* = 0.5302). Efficacy of sarolaner against an existing infestation was 86.2 and 96.9% compared with that of afoxolaner which had efficacy of 21.3 and 85.0% at 8 and 12 h time points respectively. Efficacy of sarolaner and afoxolaner reached 100% at 24 and 48 h post-treatment respectively (Table 1 and Fig. 1).

**Fig. 2:**
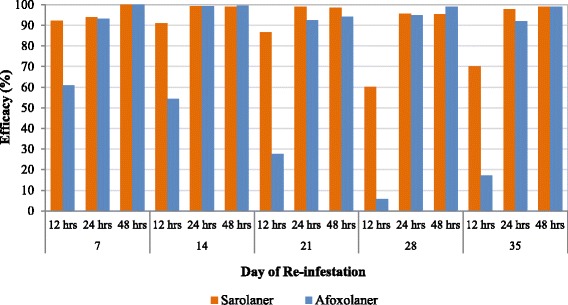
Percent efficacy based on geometric mean counts relative to placebo at 12, 24 and 48 h after weekly post-treatment re-infestations of *Ixodes holocyclus* for dogs treated with a single oral dose of sarolaner or afoxolaner on Day 0

Against subsequent weekly infestations at the 12 h time points, treatment with sarolaner resulted in significantly lower GM tick counts compared to both placebo- (*P* ≤ 0.0048) and afoxolaner-treated dogs (*P* ≤ 0.0077) on all Days (7, 14, 21, 28 and 35). Efficacy of sarolaner at the 12 h time points following weekly re-infestations ranged from 60.2 to 92.2% compared to 5.8 to 61.0% in the afoxolaner treated dogs (Table 2 and Fig. 2).

Against subsequent weekly infestations at the 24 h time points on days 22 and 36, sarolaner-treated dogs had significantly lower GM tick counts than afoxolaner-treated dogs (*P* ≤ 0.0356). On Days 8, 15, 22, 29 and 36, treatment with sarolaner and afoxolaner resulted in significantly lower GM tick counts than placebo (*P* ≤ 0.0001). At 24 h following weekly re-infestations, efficacy of sarolaner ranged from 93.9 to 99.2% compared to 91.9 to 99.2% in the afoxolaner-treated dogs (Table 3 and Fig. 2).

Against weekly infestations, at the 48 h time points treatment with sarolaner and afoxolaner resulted in significantly lower GM tick counts than placebo (*P* ≤ 0.0001) and efficacy (GM) of sarolaner and afoxolaner treated dogs were ≥ 95.5% and ≥ 94.1% respectively (Table 4 and Fig. 2).

## Discussion

A single dose of sarolaner resulted in the rapid reduction of an existing infestation of live *I. holocyclus* ticks with an efficacy of 86.2% at 8 h and 96.9% at 12 h post-treatment and the rapid kill of re-infestations for 5 weeks after treatment within 12 h of attachment with an efficacy of  ≥ 60.2%. Although the onset of tick paralysis typically does not occur until day 4 or 5 after tick attachment, the sooner an attached tick can be killed, the lower the risk of paralysis [3, 17, 19], especially those pre-existing ticks that have already been on the dogs for 3–4 days prior to treatment. The rapid speed of kill of sarolaner as early as 8 h against an existing infestation will provide faster removal of attached ticks, thereby minimising the risk of tick paralysis. Similar faster speed of kill of sarolaner has also been demonstrated against other *Ixodes* tick species such as *Ixodes scapularis* and *Ixodes ricinus* [30, 31]. The faster speed of kill and persistent efficacy of sarolaner will provide excellent overall efficacy against *I. holocyclus* for up to 5 weeks.

## Conclusions

Sarolaner had a significantly faster speed of kill at both 8 and 12 h compared with afoxolaner against existing infections of paralysis tick and had higher efficacy than afoxolaner at 12 h after re-infestation over 35 days.

The rapid and consistent kill of ticks provided by sarolaner within 24 h after a single oral dose and following weekly re-infestations over 35 days suggests this treatment will provide highly effective, rapid and reliable control of ticks over the entire treatment interval, thereby minimizing the risk of tick paralysis in dogs.

## Additional file

Additional file 1: Comparative *Ixodes holocylcus* individual tick counts. (XLSX 21 kb)

## Abbreviations

AM: Arithmetic mean
GM: Geometric mean
WAAVP: World Association for the Advancement of Veterinary Parasitology
